# A Method of Reducing Old Colles’ Fractures

**Published:** 1907-09

**Authors:** 


### A METHOD OF REDUCING OLD COLLES' FRACTURES.

McWilliams advises the following method of Elliott's for the reduction of old Colles' fractures: A large engineer's monkey wrench is produced and the blades are well padded. The patient is anesthetized and the blades so placed on the wrist that they are entirely proximally sutuated to the fracture line, the edge of the dorsal blade resting against the posteriorly projecting upper edge of the lower fragment. The blades are then screwed together, so as to fairly tightly embrace the tissues. With the expenditure of very little force, a refracture through the old fracture line may readily be produced by twisting the wrench so as to flex the lower fragment on the upper. There is no strain whatsoever brought upon the structures of the wrist itself, which consequently remains uninjured. The damage to the tendons by the blades of the wrench is trifling and is soon recovered from. The after-treatment is similar to that which obtains in cases of fresh Colles' fractures. Anterior and posterior wooden splints are used, which do not extend further than to the metacarpophalangeal joints, affording free play for the fingers. Three weeks is usually ample time to maintain immobilization in the splints. This method in almost all cases will do away with the necessity for any cutting operation.—Medical Record, May 25th, 1907.

